# Diphtheria and Membraneous Croup, Their Differential Diagnosis and Treatment

**Published:** 1898-07

**Authors:** Wilson T. Davidson

**Affiliations:** Belton, Texas


### Diphtheria and Membraneous Croup, Their Differential Diagnosis and Treatment.

BY WILSON T. DAVIDSON, M. D., BELTON, TEXAS.

(Read before the Bell County Association of Physicians and Surgeons, April 19, 1898.)

Of all conditions which confront the practitioner of general medicine, I cannot think of anything which is calculated to bring greater anxiety to the parents and to call upon the attendant for more skillful attention than the symptoms brought about by the formation of a membrane in the region of the larynx. I do not propose this afternoon to speak of diphtheria in general,—its cause, changes of structure and function, etc., but only that variety of this disease, which on account of its intimate anatomical association with what is ordinarily called "membranous croup" requires a discriminating differential diagnosis from that condition. I am not one of those who consider all forms of membranous inflammation of the larynx as diphtheria. I believe there is a specific inflammation of the larnyx not caused by the Klebs-Loeffler bacillus, and that in all probability, this germ causing croupous laryngitis is frequently associated with the streptococcus pyogenes and pneumococus as a mixed infection. I am aware of the fact that most people consider diphtheria and membranous croup or membraneous laryngitis as one and the same thing. Northrup (1) of New York, says that we may practically consider all cases of pseudo-membranous laryngitis as diphtheria. We know, too, that Park, of Boston, found positive proof of diphtheria in 80 per cent. of laryngeal cases. No doubt a great many cases of laryngeal diptheria are wrongly diagnosed as membranous croup, particularly so where there is no epidemic of diphtheria. I know it would be better to err on the side of safety, but, in doing so, we should not go so far as to say that all cases of membranous inflamation of the larynx are diphtheria. For I have seen quite a number of cases of what I would call croupous laryngitis and believe that it is a disease separate and distinct in itself.

### DIFFERENTIAL DIAGNOSIS.

Let us take some of the points of distinction between this disease and diphtheria:

1. Croupous laryngitis does not occur as an epidemic. I have known it to occur in a house where diphtheria had never been known to exist previously, and at a time when there were no cases of diphtheria in the county. Diphtheria, as we know, can usually be traced to some previous source of infection.

2. Croupous laryngitis is not usually followed by other cases. The contagious nature of diphtheria is well known.

3. In croupous laryngitis the membrane is thin, of a bluish-white or yellowish color and easily detached without presenting a bleeding surface. In all those cases of diphtheria which I have seen the membrane was a sort of mouse-color, hard to detach, revealed a bleeding surface when removed, and, if allowed to remain, presented evidences of necrosis at the end of thirty-six hours.

4. Coupous laryngitis does not present the evidences of profound sepsis, usually found in laryngeal diphtheria,—the pulse is not so weak—albuminuria is the exception rather than the rule, and upon recovery we do not have the paralitic sequelae to contend with as we do in dipptheria.

5. In all cases of true diphtheria the Klebs-Loeffler bacillus is found. If it is not found it is probably due to some fault in staining. In croupous laryngitis the ordinary micro-organisms of infection are usually found.

---

(1) American Text Book of Applied Therapeutics.

Besides this form of membranous laryngitis of which I have just been speaking, there is another form which sometimes takes place, but of which there is rarely any trouble in diagnosis. I refer to a membranous inflammation of the larynx from traumatism, such as caustic potash, sulphuric acid, chlorine gas, etc. On account of their ætiology there is rarely any mistake in diagnosis of these cases.

TREATMENT:

*Croupous Laryngitis.*—The child should be put to bed, and fed on a nourishing liquid diet. Mercury seems to have more influence on the disease than anything I have ever tried. It seems to control the extension of the membrane and serous exudation, I suppose through its antiphlogistic action. I would give calomel every two hours for the first twenty-four hours, or, better still, calomel inhalations every hour or so till its full physiological effect is brought about. The throat should be cleansed every three or four hours with some mild antiseptic solution, such as bichloride 1 to 5000, or Dobell's solution. Should the symptoms of dyspnea become more and more pronounced and threaten death by suffocation, one has a choice between tracheotomy and intubation, and will probably take the one or the other, according as his individual experience has prejudiced him in its favor. I know intubation is practiced by many of our best men, and it seems to me there are many things to commend it,—no hemorrhage, no shock, the consent of the parents is easily gotten, etc,—but, as I have never had any experience with the intubation tube, I should do tracheotomy. Emmet Holt, of New York, says that every case of membranous laryngitis should be given a dose of antitoxin. Where one is in doubt, in order to be on the safe side, I can see no objection to this procedure.

*Diphtheritic Laryngitis.*—The general therapeutic measures just enumerated should also be carried out in laryngeal diphtheria. Additional symptoms may arise later on in the disease, such as weak pulse, albuminuria, etc., and they should receive appropriate treatment. The antitoxin treatment of diphtheria has been given a thorough test by the profession and I believe it has come to stay. Dr. Welch (1) of Johns Hopkins' University, has collected 7166 cases of diphtheria treated with antitoxin

---

(1) Bull. Johns Hopkins' Hospital, July-August, 1895.

with a mortality of only 17.3 per cent. Of these cases 4294 were laryngeal and gave a mortality of 18.3 per cent. The Commission of the American Pediatric Society (1) have collected 5974 cases, with a mortality of 12.3 per cent, and, excluding those that were practically moribund, or dying within twenty-four hours, the mortality is brought down to only 8.8 per cent. The Society recommends: 1. Dosage.—For a child one year old, in laryngeal cases 1500-2000 units for the first injection, to be repeated in eighteen to twenty-four hours, if no improvement, a third dose if necessary. Severe cases in children under two years and mild cases over that age, 1000 units. Repeat if necessary. 2. Quality.—Most concentrated preparation. 3. Time.—As soon as clinical diagnosis is made.

It will be noticed that the death rate given in laryngeal cases is very low, compared to what it was before the use of antitoxin. It is in this form of the disease that antitoxin is most useful. From a close study of the report of the commission appointed by the American Pediatric Society, that of Professor Welch, of Johns Hopkins, and of several New York Infant Asylums, one is led to look npon antitoxin as a specific. The earlier it is given the less the mortality; and there is practically no mortality among those children given an immunizing dose during an epidemic.

DISCUSSION.

Dr. J. S. McKelvey: Dr. Davidson gave a very exhaustive diagnosis from a clinical standpoint, but it should also be microscopical. The diagnosis cannot be relegated entirely to the microscope, but its aid is very essential. A laboratory for making cultures is a great deal of expense, therefore one should examine the membrane; and when the clinical and microscopical coincide you can be sure. One is liable to make a mistake,—the usual form of diphtheria baccillus and bacterium sputigium cannot be absolutely differentiated. The sputigium is not in such great numbers as the Klebs-Loeffler. In croupous tonsillitis there are no bacteria in great numbers that resemble the Klebs-Loeffler. Then one can be sure when the clinical symptoms bear out the microscopical diagnosis. In regard to treatment, it should be both *antitoxin* and *general*. Give antitoxin immediately. In addition, in diphtheria, I would advise an ice-bag to the throat and a spray. Nothing else need be done except on

(1) Gould's Year Book, 1897.

a general plan. In the mixed form of diphtheria antitoxin does not have the effect it has in the pure. Has remarkable effects in the pure form. The only bad effects are a rash and rheumatic pains.

Dr. J. M. Frazier: Dr. McKelvey, what about croupous cases?

Dr. McKelvey: German authorities lean to the opinion that when a membrane is formed it is diphtheria. A germ is found in what is called pseudo-croup which is equivalent to the diphtheria bacillus in all respects except that it is mild. In croupous inflammation the membrane does not go as deep as in diphtheria.

Dr. Frazier: From the standpoint of a general practitioner, I am skeptical as to croup. Have never seen a case. Have had eight or ten sporadic cases of diphtheria. From a clinical standpoint, I don't believe there is any difference. I think they all ought to be reported as diphtheria, and antitoxin used in even suspected cases. Have never had occasion to use antitoxin; would use it if I had a case. It would be almost criminal not to use it. The doctor's paper is excellent, and I want to commend him for the manner in which he presented the subject.

Dr. Taylor Hudson: Dr. Frazier, those cases that recovered, did they have any sequelae?

Dr. Frazier: One had partial paralysis.

Dr. J. M. Woodson: I have never made a diagnosis of membranous croup. They bear about the same relation to each other that varricella does to small pox. Nothing has been said yet as to the transmission of diphtheria. Laryngeal diphtheria is less liable to transmission than any other form. There is no evidence that infection is carried by the breath. Can't see what good comes from local applications. Impossible to get any preparation in the larnyx unless during inspiration. Only way is a spray. Unless pharyngeal or retro-pharyngeal, I can't see any good from local applications. If in doubt as to diagnosis give treatment for diphtheria,—antitoxin and mercury. Give plenty of mercury; also stimulate well—gr. XXIV of mild chloride, and eight ounces of whiskey in twenty-four hours. Will give you my experience with antitoxin. Had fifteen cases of pharyngeal type, treated with antitoxin, and all recovered except one. The nasal type of the disease is especially virulent, for the lymphatics here are especially abundant. One cannot always depend upon the membrane; there is no characteristic membrane. Last year I had an experience with laryngeal diphtheria. Could see no membrane. Respiration not more labored than in ordinary catarrhal croup. My patient died that night, and at autopsy the laryngeal form of diphtheritic membrane was disclosed. Another case similar: was taken at ten o'clock, two miles from the other case. Often wonderful how these cases develop here and there. Agreed on laryngeal diphtheria, and gave treatment according to diagnosis,—three inoculations of antitoxin,—1st, 1000; 2nd, 1000, and 3rd, 500 units. Result, fatal in 36 hours. Three other children in the family were given immunizing doses and isolated. One contracted the disease. In the first family two other children received immunizing inoculations and escaped contraction of the disease. Last year I was called to Killeen. There were four children in this family, and the three healthy ones had been nursing the sick one. Soon after I got there, the child coughed up a cast of the larynx. A diagnosis of diphtheria was made. Sent the specimen to Galvestion, and Professor Allen J. Smith found the Klebs-Loeffler bacilli in abundance. At this time I thought the child convalescent, and did not advise antitoxin. Convalescence was slow. No other cases in this family. The children were isolated. They had been going to the neighbors frequently, and playing with the babies. No other case followed this case. Why there was no epidemic I can't understand. Accept it as a fact without knowing why.

Dr. Frazier: How long had the child with laryngeal diphtheria been sick when you gave the antitoxin?

Dr. Woodson: 14 hours.

Dr. Hudson: How long did it live?

Dr. Woodson: About five hours after the last dose. The child was about two years old.

Dr. U. H. Nixon: If Koch's law is correct, how can we explain the fact that the Klebs-Loeffler bacillus is found in other diseases, and in the mouth which is not diphtheritic? It must not be the cause of diphtheria.

Dr. McKelvey: At present, all authorities I have investigated deny the Klebs-Loeffler bacillus in health. It is found in the throat in many cases for three or four months. You can inoculate an animal from this source two months afterwards. There are several bacteria in the healthy mouth that resemble the diphtheria bacillus. It is hard to distinguish bacteria as a rule by microscopical examinations alone.

Dr. S. M. Jenkins: I have had but little experience with diphtheria. Most of the cases I saw died, notwithstanding the antitoxin. Was room to doubt diphtheria in those cases where antitoxin scored such a grand victory. Am skeptical as to benefits derived from antitoxin. Don't doubt cases that have gotten well. In my opinion the greatest benefit is to be derived from the mild chloride and antiseptic washes. But little faith in antitoxin.

Dr. Woodson: Don't understand from cases that I gave that I am not in favor of antitoxin. It is as nearly a specific as anything we have. Mortality has been reduced from 37.43 per cent. down to 11.15 per cent. in statistics I have read. When should we consider it safe to release a patient?

Dr. J. M. McCutcheon: A majority of authorities are undecided as to the point the doctor has just brought up. I believe there is an inflammation of the phyrnx that is not necessarily caused by the Klebs-Loeffler. Membranes are very much alike. Treatment should be same in both. Applications not too strong. I believe croup is non-infectious. Diphtheria can usually be traced to bad drainage somewhere. Many cases called croup are diphtheria, and vice versa.

Dr. J. D. Law: I know it is orthodox to eliminate membranous croup from our diagnosis altogether, but I believe there is a non-specific, non-contageous membranous croup. I think Dr. Woodson saw a case of membranous croup at Killeen. Our bacteriologists claim virulent and non-virulent forms of the Klebs-Loeffler. They are found in healthy throats. It is true that diphtheria is very infectious. I should not use antitoxin. Reduction due to follicular pharyngitis and laryngitis being called diphtheria. Just as though cases of dengue were classed as yellow fever, the mortality in yellow fever would be brought down. In regard to treatment, I agree with all that has been said except as regards antitoxin. If it is specific, why resort to other remedies? Why use tincture of iron and mercury? In four or five years I don't believe it will be used at all. I doubt the Klebs-Loeffler bacillus as the cause of diphtheria.

Dr. McKelvey: In the healthy mouth there is a bacillus which resembles the Klebs-Leffler, but it will not produce the symptoms of inoculation as does the real Klebs-Loeffler. Investigations disclose the following differences: (a) It takes the pseudo-diphtheria bacillus longer to develop on agar. (b) It turns the agar acid, while the real Klebs-Loeffler turns it alkaline. (c) The difference of inoculation symptoms.

Dr. D. L. Cummins. I have had one case of membranous croup. Called at 12 m. and patient died that night. Have not treated any cases of diphtheria.

Dr. Nixon: In the western part of our county there are a great many cases of diphtheria that die. They die with symptoms of blood-poisoning. There is a pseudo-membrane of yellowish white color. Have had ninety-four cases of follicular tonsillitis with no mortality. Let me tell you a little simple remedy that cures them: sulphur on the throat and a little aconite.

Dr. Cummins: I have been treating such cases (follicular tonsilitis) all winter. Give calomel and aconite.

Dr. Hudson: Dr. Law's ideas are nearer what I believe, than anything that I have heard, but I do believe in the Klebs-Loeffler bacillus. Am not wedded to antitoxin, but would use it. All of us now have cases of follicular tonsilitis. There are three places in Bell County where the malignant form of this affection occurs. As to diagnosis, I regard diphtheria and what is known as pseudo-membranous croup as two different and distinct diseases, due to different infections, and each having its own peculiar symptoms and pathology. Given two different cases: we are called to see a case. History;—has been sick two or three days, symptoms not severe enough to alarm parents. Supposed it had false croup, gradually grew worse, all simple remedies exhausted, physician summoned, child probably has croupous breathing, paroxysms worse,. ranging from ten to forty minutes apart, no mucus in the throat, slight elevation in temperature, no deposit in the throat, fauces or soft palate, glandular involvement, if any, very slight, some cyanosis, anxious expression of countenance—peculiar dry whistle to the breathing.

Symptoms point more to an infiltration of the sub-mucous tissue and consequent stricture along the air passage. This is what I understand to be pseudo-membranous croup. Second, called to see patient, breathing bad and more constantly so, no marked paroxysms of worse breathing at times, and deposit on tonsils probably on soft palate and fauces may be in nasal track, mucous in throat, glands of neck and throat swollen, as well as possibly sub-maxillary and sub-lingual, fever higher, symptoms alarming, face swollen, eyes and nose running, possibly some suppression of urine. This is what I would class as diphtheria.

Treatment in the former supportive and expectant; in the latter mercury, supportive and antiseptics.

Dr. McCutcheon: One point can't be too forcibly impressed, we can't be too strict in isolation. Diphtheria is very fatal, i. e., diphtheria. If it is croup, there is no harm done.

Dr. Frazier: There should be as complete isolation in this disease as in small pox. Thorough antiseptic precaution should be taken, and if the patient die everything should be burned that can be disposed of. This is important. It is a duty the physician owes the community.

Dr. Law: This is a practical point. We are derelict in our duty if we do not attend to it; yet how many physicians in Bell county are derelict in this duty? Why? Because they think it non-contageous and non-specific. I don't want you gentlemen to think I have gone back on bacteriology altogether; yet, in diphtheria, I am a little skeptical.

Dr. Davidson: I am glad that what I have had to say has brought forth such an ample discussion. In times gone by everyone acquiesced in the classification of a certain group of symptoms as membranous croup. In recent years there is a disposition to classify all membranous inflammations of the throat as diphtheria. In my opinion the pendulum has swung too far to one side. If an error be made, it should be on the safe side and treatment given for diphtheria. The existence of non-virulent forms of the Klebs-Loeffler is an undisputed fact; that this germ may become unusually virulent is equally certain. An explanation of these facts would lead us into a prolonged research in the fields of biology. One of the gentlemen asked how it is, if Koch's law be true, that we may have the Klebs-Loeffler bacillus in the healthy throat. It is not merely the presence of the germ,—there must be an inoculation,—to produce diphtheria. Catarrhal inflammations of the throat predispose to this inoculation. In extensive epidemics of diphtheria many people are found to have a slight inflammation of the throat in which the Klebs-Loeffler bacillus is found. This is really diphtheria, but in a very mild form. A possible explanation of the circumstances that the laryngeal form of diphtheria is not so readily communicated to the children of the same household may be found in the fact that there is not so much mucous and saliva cast off as in those forms higher up. I believe the entire profession will eventually fall into line in the use of antitoxin. It comes as near being a specific as anything I know of. Has already taken a place right along side of quinine in malaria and mercury in syphilis. The fact that we do not exactly understand its mode of action, should not prejudice us against it. Vaccination has saved millions of human lives, and we do not yet know exactly how immunity is brought about. The principle of serum therapy in all diseases of microbic origin is the same. Look at the results attained by the Pasteur institutes for the treatment of rabies. As soon as the yellow fever germ is definitely settled upon, and pure cultures made, we will have a serum for the treatment of that disease. I cannot state too emphatically that I believe it is the duty of everyone of us to be prepared to use antitoxin in all cases of diphtheria.

